# Operative Choice for Length-Unstable Femoral Shaft Fracture in School-Aged Children: Locking Plate vs. Monolateral External Fixator

**DOI:** 10.3389/fped.2021.799487

**Published:** 2022-02-10

**Authors:** Pan Hong, Saroj Rai, Xin Tang, Ruikang Liu, Jin Li

**Affiliations:** ^1^Department of Orthopaedic Surgery, Union Hospital, Tongji Medical College, Huazhong University of Science and Technology, Wuhan, China; ^2^Department of Orthopaedics and Trauma Surgery, Blue Cross Hospital, Kathmandu, Nepal; ^3^First Clinical School, Tongji Medical College, Huazhong University of Science and Technology, Wuhan, China

**Keywords:** femoral shaft fracture, children, locking plate, monolateral external fixator, length unstable

## Abstract

**Background:**

Locking plate (LP) is a good choice in the treatment of length-unstable femoral shaft fracture in children. Monolateral external fixator (EF) has been reported for this condition for decades. This study aims to compare the clinical outcomes of school-aged children with length-unstable femoral shaft fracture treated with LP *vs*. EF.

**Methods:**

Patients aged 5–11 years old with length-unstable femoral shaft fractures treated at our institute from January 2014 to January 2018 were retrospectively reviewed and categorized into LP and EF groups. The preoperative data, including baseline information of the patients, radiographic parameters, and types of surgical procedure, were collected from the hospital database, and postoperative data, including complications, were collected during the follow-up visits.

**Results:**

Overall, 36 patients (average, 8.2 ± 2.1 years; male, 20; female, 16) in the LP group and 35 patients (average, 8.3 ± 2.3 years; male 20, female 15) in the EF group were included. There was significantly less operative time for EF (45.4 ± 7.8 min) compared with LP (67.8 ± 11.3 min) (*P* < 0.001). As for the frequency of fluoroscopy, there was a significant difference between the EF (13.9 ± 2.4) and LP (16.5 ± 3.2) groups (*p* < 0.001). The rate of major complications was not significantly different between these two groups. There was a significant difference between the EF group (11.2 ± 5.8 mm) and the LP group (7.5 ± 1.6 mm) group concerning limb length discrepancy (*P* < 0.001).

**Conclusion:**

Both LP and EF produce satisfactory outcomes in school-aged children with length unstable femoral shaft fractures. External fixation remains a viable choice without the necessity of secondary surgery for hardware removal.

## Background

The treatment strategy of pediatric femoral shaft fracture has been evolving in the past years (1, 2). Traction followed by spica casting, elastic stable intramedullary nail (ESIN), plating, external fixator (EF), and antegrade rigid intramedullary nailing have been reported for the treatment of femoral fracture in children and adolescents (3–7).

ESIN has been widely used in the treatment of school-aged patients with femoral shaft fractures (2). However, for children with length-unstable fracture, plating proved to be superior to ESIN in clinical outcomes (8, 9), but the plate requires removal afterwards, a process with reported complications of hemorrhage and infection. Locking plate has been reported in pediatric population for decades, with a shorter incision than that of traditional reconstruction plate (10). Besides this, EF has been a valuable option for pediatric femoral shaft fractures for decades, but the complications, including superficial infection, difficulty of daily care, and possibility of refracture, lead to the waning enthusiasm of its application in recent years (11, 12). Nevertheless, EF could be easily removed during out-patient visits.

This study aims to compare the clinical outcomes of school-aged children with length-unstable femoral shaft fracture treated with LP *vs*. EF.

## Methods

**Figure 1:**
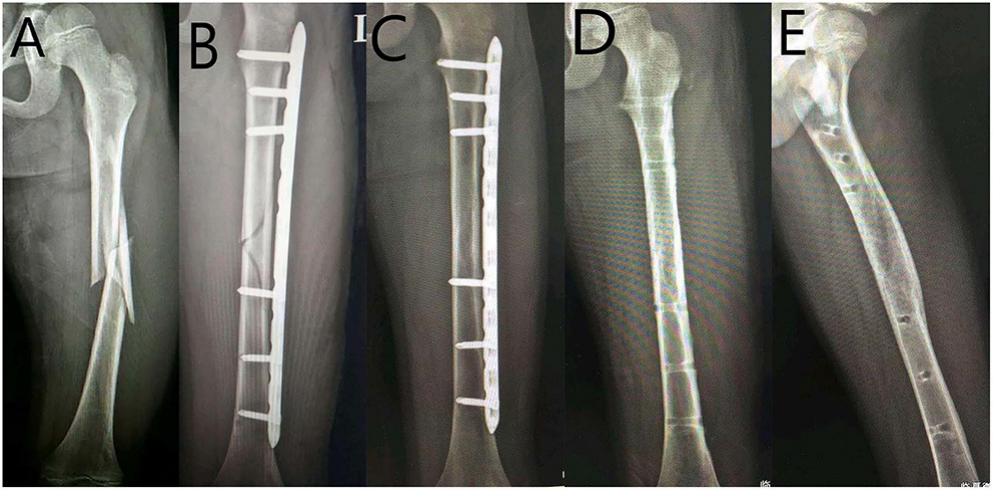
Nine-year-old girl with left femoral shaft fracture treated with locking plate. **(A)** Anteroposterior (AP) view of the left femur before surgery. **(B)** AP view of the left femur after surgery. **(C)** AP view of the left femur at 9-month follow-up. **(D)** AP view of the left femur after hardware removal. **(E)** Lateral view of the left femur after hardware removal.

**Figure 2:**
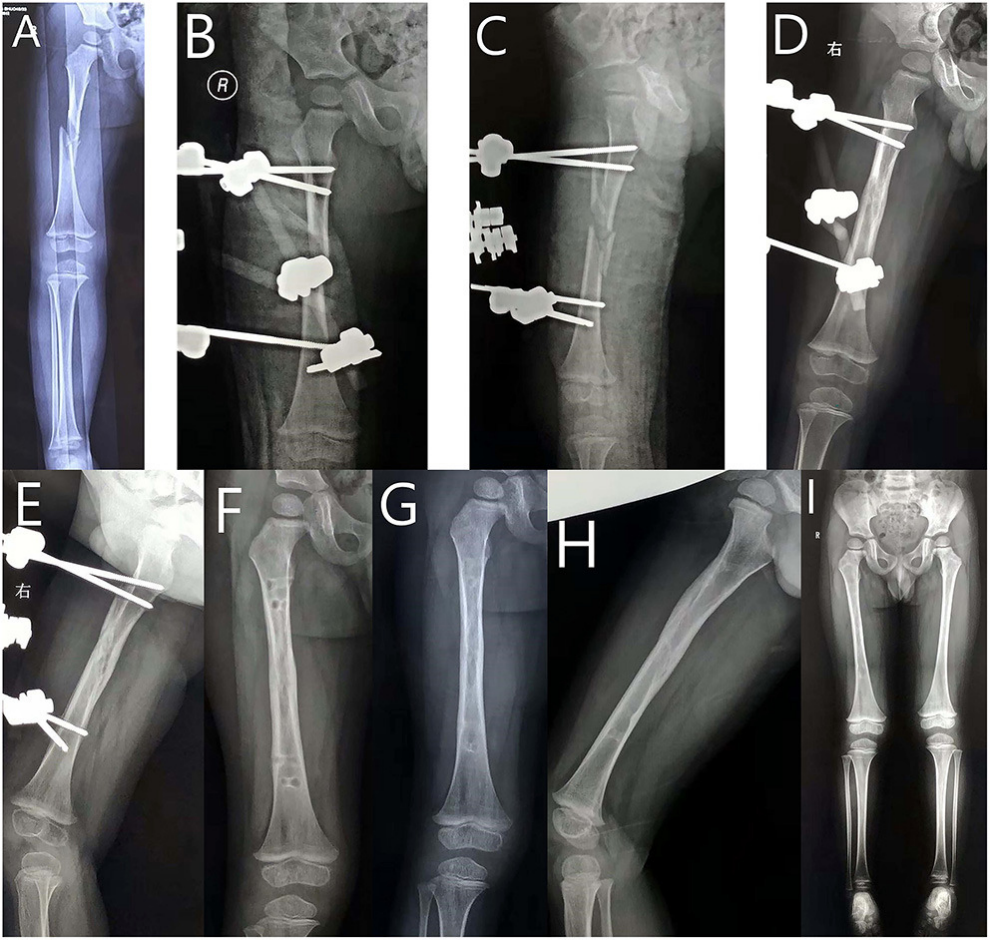
Seven-year-old boy with right femoral shaft fracture treated with an external fixator. **(A)** Anteroposterior (AP) view of the right femur before surgery. **(B)** AP view of the right femur after surgery. **(C)** Lateral view of the right femur after surgery. **(D)** AP view of the right femur at 6-week follow-up. **(E)** Lateral view of the right femur at 6-week follow-up. **(F)** AP view of the right femur at 9-week follow-up. **(G)** AP view of the right femur at 6-month follow-up. **(H)** Lateral view of the right femur at 6-month follow-up. **(I)** AP view of the lower limbs at 18-month follow-up.

Patients aged from 5 to 11 years old who were treated in our hospital from January 2014 to January 2018 were reviewed retrospectively and were divided into LP and EF groups according to the fixation methods (see Figures 1, 2).

In our study, length-unstable femoral shaft fracture with a comminuted or long, oblique fracture line was classified as 32-D/4.2, 32-D/5.1, and 32-D/5.2 according to AO Pediatric Comprehensive Classification of Long Bone Fractures (4, 13). The exclusion criteria included patients over 50 kg in body weight, aged 12 years old or above, or with pathological fracture, open fracture, neuromuscular or metabolic diseases, previous femoral fracture or instrumentation, and without complete medical records or follow-up of <18 months (4).

The preoperative data including basic information were extracted from the hospital database. Postoperative data including complications were collected during the out-patient visits (4).

The total length of the femur was determined by anteroposterior (AP) radiography. In our study, significant limb length discrepancy (LLD) was defined as a difference of at least 2 cm between the injured and contralateral legs (3, 4). Moreover, significant angulation was defined as coronal angulation >10° or sagittal angulation >10° (4).

Callus across the fracture site on at least three out of four cortices on AP and lateral radiograph is defined as union in our study (3–5). Flynn scoring system was used to evaluate the final functional outcome at latest follow-up visit (14). Major complications included non-union or loss of reduction and deep infection, which required revision surgeries (3, 4). Minor complications included mild angular deformity, mild LLD, and superficial infection (3, 4).

Long-leg slab was used in both groups for 1–2 weeks to alleviate swelling and post-operative pain. Non-weight-bearing exercises were initiated after slab removal. Toe-touch weight-bearing was encouraged when union on X-ray was noticed during the out-patient visits, and progression to full weight-bearing was allowed according to the X-ray manifestation and physical examination.

LP was routinely removed 9–12 months after the primary surgery in the operating room. In contrast, EF was removed 6–12 weeks postoperatively at the out-patient department, followed by immobilization in a long leg brace for 3–4 weeks.

The SPSS statistical package program (SPSS 19.0 version; SPSS Inc., Chicago, IL, USA) was adopted in our study. Data are presented as mean ± standard deviation or *n* (%). *P* < 0.05 was considered statistically significant.

## Results

As shown in Table 1, 36 patients (average, 8.2 ± 2.1 years; male: 20, female: 16) in the LP group and 35 patients (average, 8.3 ± 2.3 years; male: 20, female: 15) in the EF group were included in this study. There was no significant difference between the two groups concerning the demographic parameters of the patient, including sex, age, weight, operative side, and duration from injury to surgery.

**Table 1:** Patient demographic.

**Parameters**		**LP (*N* = 36)**	**EF (*N* = 35)**	***P*-value**
Sex	Male	20	20	0.768
	Female	16	15	
Side	Left	19	19	0.467
	Right	17	16	
Age (Y)		8.2 ± 2.1	8.3 ± 2.3	0.551
Weight (Kg)		29.0 ± 5.8	29.9 ± 6.6	0.522
Injury to surgery (d)		2.2 ± 0.8	1.9 ± 0.8	0.148

Comparing the operative variables (Table 2), there was significantly less operative time for EF (45.4 ± 7.8 min) as compared with LP (67.8 ± 11.3 min) (*P* < 0.001). As for the frequency of fluoroscopy, there was a significant difference between the EF (13.9 ± 2.4) and LP (16.5 ± 3.2) groups (*P* < 0.001). The length of hospital stay was shorter in the EF group (3.0 ± 0.9 days) than the LP group (4.0 ± 0.8 days) (*P* < 0.001).

**Table 2:** Operative parameters for fracture surgery.

**Parameters**	**LP (*N* = 36)**	**EF (*N* = 35)**	***P*-value**
Operative time (min)	67.8 ± 11.3	45.4 ± 7.8	<0.001
Fluoroscopy (times)	16.5 ± 3.2	13.9 ± 2.4	<0.001
Length of stay (days)	3.0 ± 0.9	4.0 ± 0.8	<0.001

As shown in Table 3, the patients in both groups showed significantly reduced pain after surgery. There was no significant difference between the two groups concerning pain response after surgery.

**Table 3:** Pain management.

**Parameters**	**LP (*N* = 36)**	**EF (*N* = 35)**	***P*-value**
VAS before surgery	7.2 ± 0.8	7.0 ± 0.8	0.29
VAS (1^st^ day)	5.2 ± 0.7	5.4 ± 0.9	0.78
VAS (1–3 days)	3.6 ± 0.7	3.9 ± 0.8	0.23

As shown in Table 4, the rate of major complications was not significantly different between these two groups. There was a significant difference between the EF group (11.2 ± 5.8 mm) and the LP group (7.5 ± 1.6 mm) concerning limb length discrepancy (*P* < 0.001). In the frontal and sagittal planes, the angulation was higher in the EF group than in the LP group.

**Table 4:** Complications after surgery.

**Complication**	**LP (*N* = 36)**	**EF (*N* = 35)**	***P*-value**
Loss of reduction	0	0	>0.999
Non-union	0	0	>0.999
Refracture	1 (2.7%)	1 (2.8%)	0.765
LLD (mm)	7.5 ± 1.6	11.2 ± 5.8	<0.001
Frontal angulation (degree)	5.1 ± 0.9	7.4 ± 1.1	<0.001
Sagittal angulation (degree)	6.3 ± 1.6	8.3± 2.6	<0.001

*Major complications: loss of reduction, non-union, refracture*.

As shown in Table 5, the excellent rate of the Flynn score system after implant removal was significantly higher in the LP group (83.3%) compared with the EF group (54.3%), and both groups had no patient with a poor score.

**Table 5:** Clinical parameters after implant removal.

**Parameters**		**LP (*N* = 36)**	**EF (*N* = 35)**	***P*-value**
Flynn	Excellent	30 (83.3%)	19 (54.3%)	<0.001
Score	Satisfactory	6 (16.7%)	16 (45.7)	
System	Poor	0	0	
Excellent + satisfactory		36 (100%)	35 (100%)	>0.999

## Discussion

EF has the potential advantages of being a minimally invasive approach for the treatment of pediatric femoral shaft fracture, with shorter operative time and length of hospital stay and no requirement for secondary surgery. EF produces satisfactory clinical outcomes for the treatment of length-unstable femoral shaft fracture in children, and it is comparable with the LP.

Several interventions have been reported for the treatment of length-unstable femoral shaft fracture in children, including traction, followed by spica casting, ESIN, plating, rigid nailing, and external fixator with various constructs (15). Spica casting following traction requires prolonged hospital stay and demonstrates limited ability of restoring the limb length. ESIN has been reported in low-grade comminuted femoral shaft fracture (16, 17), but plating has demonstrated superiority over ESIN in children with length-unstable femoral shaft fractures (18). In children younger than 8 years old, both ESIN and EF can be considered as safe and effective choices for pediatric femoral shaft fracture (19). However, more complications were witnessed in unstable femoral shaft fractures treated with ESIN (20, 21).

Locking plate has been reported for this condition for decades, and it could be implemented in a minimally invasive approach. However, it demands a secondary operation for implant removal. Rigid nailing is a reasonable choice for comminuted fracture, but avascular necrosis of femoral head in children is a troublesome complication (22), and it is contraindicated in younger children. EF has been reported for long bone fracture decades ago, but its complications of superficial infection, scarring around the pin tract, refracture after EF removal, and serious LLD have been reported in the literature (23, 24). Besides these, the satisfactory clinical outcomes of plating lead to the waning enthusiasm for EF in closed fractures (25). However, secondary operation for plate removal made it unacceptable for the legal guardians of certain patients. Therefore, after thorough deliberation and discussion with the surgical team, some of the parents might choose EF as it produces similar clinical outcomes as shown in our study, with acceptable minor complications such as pin tract infection (PTI) and inconvenience.

In this study, almost all patients in the EF group healed uneventfully, consistent with previous reports (26–28). All patients in the EF group demonstrated <10° angulation in the last follow-up, which is not clinically significant.

The postoperative complications of EF include malunion, delayed union, refracture, and PTI (12). There was no case of malunion that required revision surgery in either group. The rate of delayed union in our study was nil in both groups. One patient in the LP group and one patient in the EF group suffered refracture because of accidental fall after the hardware removal.

Active exercises of knee joints were partly encumbered in the EF group possibly due to the thick muscle enveloping the femur and its friction between the muscle and Schanz screw. Nevertheless, the EF was routinely removed at 6–12 weeks postoperatively, and active exercises were encouraged then without impediment. LP was routinely removed at 9–12 months after primary surgery. Although both techniques are minimally invasive, there was no incision in the EF group, whereas longer incision might be required in the LP group to remove the plate and screws afterwards. Moreover, the length of hospital stay was shorter in the EF group than in the LP group.

PTI is common during the application of an external fixator (29). Still no patient in the EF group required intravenous antibiotics or supplemental surgery, consistent with previous reports (26–28, 30). Oral antibiotics alleviated the PTI effectively. Therefore, most of the children and their caretakers were able to tolerate these minor complications well. In contrast, the daily care of patients with LP is much easier. In older teenagers with a heavier body weight, LP might be a better choice as it is without the necessity of daily care like for EF in a prolonged period of healing time. As for diaphyseal comminuted fractures in the proximal or distal femur, LP might be a superior choice with its better manageability than EF.

Besides these, there was no need for another surgery under general anesthesia. The removal of plate is fraught with complications, including the difficult removal process because of ingrown bone over the plate, longer incision wound, and risk of postoperative hemorrhage and infection (31, 32).

Limb length discrepancy (LLD) is a common complication in pediatric femoral fractures (33). However, in our study, there was no case of LLD over 2 cm at the last follow-up in both groups, probably due to the limited dissection of the fracture site.

There were several limitations in our study. Firstly, it was a retrospective study with a modest sample size; therefore, our findings should be interpreted with caution. Secondly, the allocation process of patients to either the LP group or the EF group partly depended on the preference of the surgeon in charge, and this strategy may cause allocation bias. Thirdly, although most overgrowth happens within 18 months after an injury (34), the follow-up in our study was not long enough to monitor the long-term impact on skeletal growth and development. Finally, patients receiving other treatments, including traction followed by casting and ESIN combined with EF, were not included in this study to elucidate the optimal choice for this type of fracture.

## Conclusion

Both LP and EF produce satisfactory outcomes in school-aged children with length-unstable femoral shaft fractures. External fixation remains a viable choice for this condition, without the necessity of hardware removal afterwards.

## Data Availability Statement

The original contributions presented in the study are included in the article/supplementary material, further inquiries can be directed to the corresponding author.

## Ethics Statement

The studies involving human participants were reviewed and approved by Ethics Committee of Tongji Medical College, Huazhong University of Science and Technology (IORG No: IORG0003571). Written informed consent to participate in this study was provided by the participants' legal guardian/next of kin.

## Author Contributions

JL was in charge of the main idea and is the guarantor of the integrity of the entire clinical study. PH was in charge of the study concepts, design, and manuscript preparation and editing. RL and SR were in charge of the language polishing and the grammar revision. XT and PH were in charge of the collection of the data. RL performed the statistical analysis. All authors read and approved the final manuscript.

## Conflict of Interest

The authors declare that the research was conducted in the absence of any commercial or financial relationships that could be construed as a potential conflict of interest.

## Publisher's Note

All claims expressed in this article are solely those of the authors and do not necessarily represent those of their affiliated organizations, or those of the publisher, the editors and the reviewers. Any product that may be evaluated in this article, or claim that may be made by its manufacturer, is not guaranteed or endorsed by the publisher.

## Abbreviations

LP: locking plate
EF: monolateral external fixator
ESIN: elastic stable intramedullary nail
LLD: limb length discrepancy
AP: anteroposterior
PTI: pin tract infection.
